# High planktonic diversity in mountain lakes contains similar contributions of autotrophic, heterotrophic and parasitic eukaryotic life forms

**DOI:** 10.1038/s41598-018-22835-3

**Published:** 2018-03-13

**Authors:** Rüdiger Ortiz-Álvarez, Xavier Triadó-Margarit, Lluís Camarero, Emilio O. Casamayor, Jordi Catalan

**Affiliations:** 1 Integrative Freshwater Ecology Group, Center for Advanced Studies of Blanes-CSIC. Acc. Cala St Francesc 14, E-17300 Blanes, Catalonia Spain; 2 CREAF - CSIC, Campus UAB, Edifici C, 08193 Cerdanyola del Vallès, Catalonia Spain

## Abstract

A rich eukaryotic planktonic community exists in high-mountain lakes despite the diluted, oligotrophic and cold, harsh prevailing conditions. Attempts of an overarching appraisal have been traditionally hampered by observational limitations of small, colorless, and soft eukaryotes. We aimed to uncover the regional eukaryotic biodiversity of a mountain lakes district to obtain general conclusions on diversity patterns, dominance, geographic diversification, and food-web players common to oligotrophic worldwide distributed freshwater systems. An unprecedented survey of 227 high-altitude lakes comprising large environmental gradients was carried out using Illumina massive tag sequencing of the 18S rRNA gene. We observed a large Chrysophyceae dominance in richness, abundance and novelty, and unveiled an unexpected richness in heterotrophic phagotrophs and parasites. In particular, Cercozoa and Chytridiomycota showed diversity features similar to the dominant autotrophic groups. The prominent beta-dispersion shown by parasites suggests highly specific interactions and a relevant role in food webs. Interestingly, the freshwater Pyrenean metacommunity contained more diverse specific populations than its closest marine oligotrophic equivalent, with consistently higher beta-diversity. The relevance of unseen groups opens new perspectives for the better understanding of planktonic food webs. Mountain lakes, with remarkable environmental idiosyncrasies, may be suitable environments for the genetic diversification of microscopic eukaryotic life forms.

## Introduction

Hidden species richness in ultraoligotrophic freshwaters ecosystems has long been suspected as early microscopic assessments indicated abundant soft and rather small eukaryotic organisms^1^. In ecological studies, many species of recognized relevant groups (e.g. Chrysophyceae) were only tentatively determined or, quite often, lumped into size classes, due to the lack of a consistent taxonomy^2–5^. Recently, the introduction of molecular techniques is progressively unveiling a great eukaryotic diversity in mountain lakes^6–8^. The use of molecular tools for identification of sequences originating from environmental DNA by reference to sequence databases can overcome many limitations of traditional microscopic approaches^9^, although they may lack specificity to link morphology to molecular data^10^. So far, the molecular diversity of protists in inland waters appears higher than that of the morphospecies and cultivated species catalogued in public databases^11–13^.

Despite remarkable traditional attempts to develop a morphological taxonomy (e.g. studies by H. Skuja, A. Pascher, K. Starmach, P. Bourrelly among others), the realistic appraisal of the species richness across oligotrophic lakes was hampered by technical limitations. The relevance of scarcely visible organisms, usually heterotrophs, has been a matter of speculation or they have been just simply ignored. Heterotrophic flagellates (HNF) were usually amalgamated in a single functional guild, and amoeboid forms were rarely considered and, if so, with limited taxonomic resolution^14,15^. Recent findings in marine waters have revealed large unseen diversity of heterotrophic protistan groups^16^, and many studies are highlighting the role of fungi in aquatic food webs as a promising research topic^9,11,17^.

Oligotrophic lakes are widely heterogeneous regarding climatic, chemical and morphological conditions. This heterogeneity sustains the proposal that freshwater microbial diversity might be higher than in marine environments^18–20^. An appraisal of the general eukaryotic diversity in oligotrophic planktonic systems requires an extensive survey of sites comprising several environmental gradients. Pyrenean mountain lakes provide such an environmental variation because of their distribution across altitude, and bedrock diversity^21^. Their abundance in a relatively restricted territory also permits considering patterns related to the spatial distribution and connectivity between lakes. In the present investigation, we have characterised the eukaryotic regional metacommunity of the mountain lakes of the Pyrenees and evaluated its novelty using the V9 region of the 18S rRNA gene as a taxonomic indicator. Diversity, dominance, and geographic diversification of the main groups were examined. We grouped operational taxonomic units (OTUs) in taxonomic levels that responded to similar life forms and trophic roles. Thus, some general conclusions on diversity patterns, ecological structure and food-web players were unveiled.

## Materials and Methods

### Lakes survey

**Figure 1:**
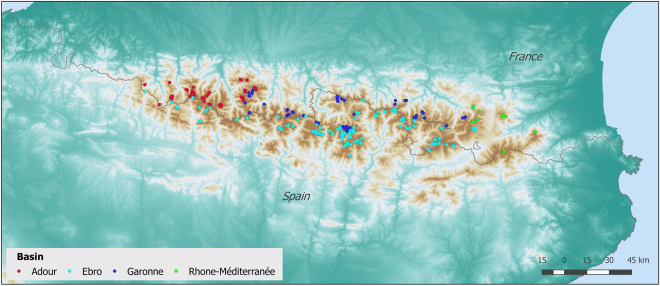
Distribution of 227 lakes surveyed and sequenced across the Pyrenees. The main river basins are indicated. Dots of nearby lakes may overlap. Map created with ArcGIS 10.1 (©ESRI).

The lakes sampling design covered the whole geographical distribution of the mountain lakes in the Pyrenees, from 0°42′21.6′′W to 2°27′45.9′′E longitude and 42°25′10.0′′N to 42°56′06.4′′N latitude (Fig. 1). The 227 sampled lakes were distributed across altitudinal gradient (1459–2990 m above sea level; average 2300 m) and different bedrock types to encompass the main environmental gradients influencing the species distribution in mountain lakes^21^. Lake area ranged between 0.1 and 57 ha (average 4.6 ha). We intended to sample all lakes within a selected sub-basin to accurately mimic the lake size, and hydrological connectivity distributions. Most of the lakes were located in the alpine belt where bare rock and scree predominated, with 40% soil coverage by meadows and shrubs on average. The predominant bedrock was granodiorite, which shaped highly diluted waters (average conductivity 33 μS/cm). Additional areas of limestone and metamorphic rocks (schist and slate) provided a broad range of chemical conditions (e.g., pH range 4.4–10.1, average 7.2). Typically, the lakes were poor in phosphorus (average soluble reactive phosphorous 73 nM), and mostly ranked as oligotrophic^22^. Plankton samples were collected in the littoral zone along the summer period to keep the temporal interval the shortest as possible and to preserve comparability among samples. About 200 mL <50 μm size water lake samples were filtered *in situ* using 0.22-micron Sterivex pressure driven sterile filter units (Millipore R), and preserved in lysis buffer (40 mM EDTA, 50 mM Tris, pH 8.3, 0.75 M sucrose) as recently reported^23^. For the genomic DNA extraction, the membranes were enzymatically digested with lysozyme, proteinase K and sodium dodecylsulfate incubation, followed by phenol extraction, and DNA purification and concentration with Amicon® Ultra 4 Centrifugal Filter Units – 100000 NMWL (Millipore).

### Sequencing and data filtering

High-speed multiplexed 18S rRNA gene sequencing with the Illumina MiSeq System (2 × 150 bp) was carried out with the “universal” 1391 f and EukBr eukaryotic primers (V9 region)^24^. These primers are known to recover the known eukaryotic diversity without major qualitative or quantitative biases^16,25^. Guidelines from the Earth microbiome project (EMP) protocol, and the genomics core facilities and methods of the RTSF-MSU (Michigan State University, USA) were followed. Raw sequences were analysed with UPARSE^26^. Overall quality of sequences was high, with 77.6% reaching Q30. The total number of sequences before quality filtering was 12,288,923. After merging of read pairs and filtering by read length (above or equal to 161 pb), quality-score distribution (ASCII ‘B’) and an expected error of 0.25, we kept 6,384,407 sequences. Out of them 921,749 were unique and 205,694 not singletons. The latter were clustered at 97% identity, after chimera removal, resulting in 6398 OTUs. Sequences were mapped back into OTUs and classified with the SILVA 119 database^27^. Overall, we retrieved a mean of 24,428 sequences per lake (range 10677–45940).

### Ecological classification and diversity indexes

Sequences were classified using the RDP classifier by default as implemented in Qiime^28^ and the Silva-NGS pipeline with the Silva 119 database^29^. Relevant discrepancies were double-checked through local BLAST (search September 2015), and if not resolved left as “Unclassified”. Around 7% of the OTUs could not be classified beyond “domain”. Mean number of OTUs per lake (Richness) and average occurrence of OTUs were determined. Shannon diversity^30^ per lake (alpha diversity), Shannon diversity in the whole region (gamma diversity) and Berger-Parker dominance^31^ indexes were calculated.

For classification *ad hoc* taxonomical levels reflecting the main ecological life-styles were used. Furthermore, preferences for carbon source, nutrition type and habitat were labelled. Thus, groups corresponded to distinct taxonomic hierarchical levels but shared the common characteristic that most of the species within the group showed a similar life form and functional role within the planktonic food web. For the first ten most relevant groups, we evaluated in detail more different diversity patterns (Table 1).

### Genetic novelty analysis

The novelty in the dataset was explored by 18S rRNA gene BLAST identity searches against GenBank sequences (search September 2015) to both the closest environmental match (CEM) and the closest cultured match (CCM) available in GenBank (e.g., Massana *et al*.^32^). Only OTUs sharing sequence identity values and alignment coverage values above 80% to nt database were considered for downstream analyses. The closest match identity values (either to environmental or cultured) were used to explore novelty-abundance, and novelty-occurrence relationships. High novelty was defined for those OTUs with <97% identity for both CCM and CEM (see more details in Supplementary information S1).

### Geographic patterns and beta-dispersion

The relationship between geographic Euclidean distance and community dissimilarity (Bray-Curtis) was evaluated using Mantel tests (R package vegan) with the Spearman method both for the whole eukaryotic assemblage and for taxonomic groups of interest. We assigned lakes to basins and sub-basins using the ‘Spatial Join’ routine from ArcGIS 10.1 (©ESRI). We estimated average beta-dispersion (i.e., average distance to group centroids) (R package vegan) per catchment or sub- catchment, and tested it with ANOVA (999 permutations) using the taxonomic groups as levels of a factor ‘taxonomy’. If significant, *post hoc* pairwise comparisons or HSD Tukey tests were carried out to find differences between groups (R package agricolae). The coherence between pairs of taxonomic groups regarding how beta-dispersion changed in sub-basins was assessed using Spearman correlations (R package base).

## Results

**Figure 2:**
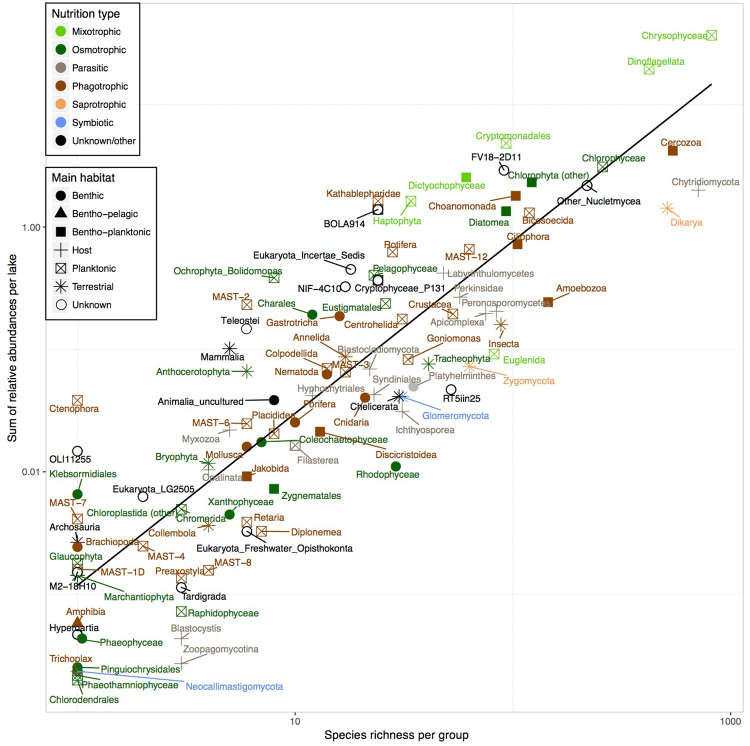
OTU richness and cumulative relative abundance of the main groups found in the plankton of Pyrenean lakes in logarithmic scale. Colours indicate dominant nutrition type while shapes indicate their main habitats. Autotrophs are split into two groups: those purely osmotrophs and those also able of phagotrophic ingestion (mixotrophs). A fitted linear model is drawn.

The total number of properly identified OTUs reached 6086 (mean 303 per lake, range 82–715), comprising 105 differential groups (Fig. 2). Most of the metacommunity richness corresponded to planktonic (37%) and bentho-planktonic (20%) free organisms, followed by host-dependent organisms (18%). The remaining component were putative zoospores, diaspores and traces of predominantly terrestrial (11%) and benthic (3%) organisms. The most abundant were autotrophic organisms (73%), but most of them (63% of the total relative abundance) were mixotrophs –i.e., autrotrophs that also ingest prokaryotes– and only a minority (10%) exclusively osmotrophs. Phagotrophic heterotrophs accounted for 12% of the reads, while other heterotrophic life-forms were less abundant (c. 3–0.1%). However, most of the OTUs richness belonged to heterotrophs: phagotrophs (26%), parasites (18%), saprotrophs (9%), and symbiotic (0.5%). Autotrophic organisms accounted for 36% of the richness (mixotrophs, 25%; exclusively osmotrophs, 11%) and trophic mode was unknown for the remaining OTUs. Overall, there was a general positive relationship between abundance and richness of the groups (Fig. 2).

Taxonomically, the regional pool was dominated by phytoplanktonic mixotrophic flagellates of Chrysophyceae (37% of the sequences and 818 OTUs), followed by Dinoflagellata (20%, 422 OTUs), Cryptomonadales (5%, 93 OTUs), and Haptophyta (2%, 34 OTUs). Among exclusively osmotrophic phytoplankton, Chlorophyta were the most abundant and diverse (5% and 378 OTUs), particularly Chlorophyceae, including also many flagellated forms. Diatomea showed relative abundance slightly above 1% of the sequences with 93 OTUs.

Regarding the motile heterotrophic components of the samples, the pseudopodia-feeding group Cercozoa (Rhizaria) with 4.2% of the sequences and 542 OTUs was the most abundant and diverse. Other groups above 1% of the sequences were Choanomonada, Kathablepharidae, and Bicosoecida. Ciliophora were slightly less abundant than these groups but with substantial richness. In contrast, Kathablepharidae and Cryptophyceae showed poor OTUs richness. Among mycoplankton, the mostly parasitic fungi Chytridiomycota (2% relative abundance) showed high richness (710 OTUs). Mainly terrestrial Dikarya, including Ascomycota and Basidiomycota (511 OTUs), and Zygomycota (63 OTUs) were probably present as dispersal stages.

### Eukaryotic genetic novelty in the Pyrenean lacustrine district

**Figure 3:**
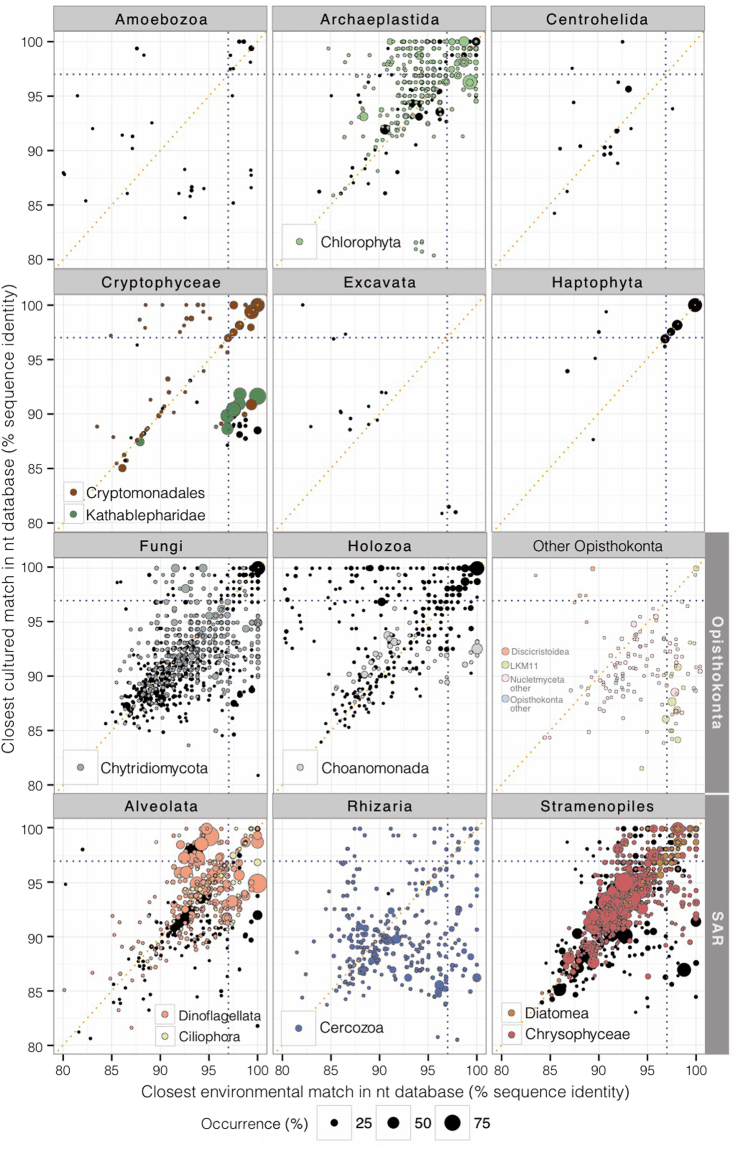
Novelty of OTUs as indicated by their closest cultured and environmental matches identity values. The highest novelty area is indicated, and falls below the 97% thresholds. Color displays the main groups of study, leaving other OTUs of the high taxonomic ranks in black. Dot size indicates OTU occurrence in the dataset.

As a common trait, large proportions of highly novel representatives were observed for the different taxa. The main major groups SAR (Stramenopiles-Alveolata-Rhizaria), Opisthokonta, and Archaeplastida, showed 81%, 74% and 54% of the OTUs within the highest novelty, respectively (Fig. 3). This value raised to >90% in the minor groups Centrohelida and Excavata. SAR had the largest diversity of both autotrophic (Chrysophyceae) and heterotrophic (Cercozoa) groups and also the greatest novelty. Chrysophyceae were mainly recovered within the highest novelty plot area, equally distant from environmental and cultured references even for those OTUs showing the highest occurrence. Numerous Rhizaria, which essentially corresponded to Cercozoa, were far below the 97% threshold. Within Opisthokonta, it was remarkable the extremely high novelty in Fungi. Indeed, most of the Chytridiomycota OTUs, true aquatic fungi, were in the highest novelty area of the plot and the mean value of the closest match was substantially low (Table 1). A smaller set of OTUs related to potential basal fungi (LKM11 and LKM15) previously found in environmental studies, showed low relatedness to cultured counterparts. Choanomonada also showed a great degree of novelty, although the most common OTU had been previously reported in environmental surveys. Photosynthetic Chlorophyta and Cryptophyceae showed close cultured references. Conversely, the heterotrophic group of Cryptophyceae (Kathablepharidae) lacked close cultured counterparts (<93% identity to CCM). Finally, Dinoflagellata and Haptophyta showed low novelty. In general, the relationship between the degree of genetic novelty and both abundance (r = 0.21, p < 0.01) and occurrence of the OTUs (r = 0.23, p < 0.001) was low. Thus, the most abundant and frequent OTUs were not necessarily previously known.

**Table 1:** Regional (lacustrine district) and local (per lake) diversity indexes.

Group	Regional parameters				Local parameters		
	Closest match (%)	Mean OTU occurrence	α-diversity (OTU Nr)	γ-diversity (H′)	α-diversity (OTU Nr)	α-diversity (H′)	BP dominance (%)
Diatomea	97.48	4.29	93	1.98	4.01	0.62	0.58
Cryptomonadales	96.95	6.47	93	1.36	6.04	0.6	0.54
Ciliophora	96.89	2.96	105	2.64	3.12	0.7	0.33
Kathablepharidae	96.89	23.46	24	0.86	5.65	0.47	0.79
Chlorophyta	96.87	3.51	378	3.26	11.86	1.17	0.12
Dinoflagellata	95.60	6.25	422	2.79	26.53	1.4	0.21
Chytridiomycota	93.71	1.92	710	4.93	13.73	1.7	0.1
Chrysophyceae	93.63	9.19	818	3.88	75.53	2.29	0.12
Choanomonada	92.48	3.83	103	2.41	3.96	0.58	0.45
Cercozoa	91.98	2.49	542	3.81	13.56	1.51	0.16

The mean closest match identity and OTU occurrence, Alpha and Gamma diversities estimated by the total number of OTUs (Richness, OTU Nr), Shannon diversity index (H′), and Berger-Parker dominance index (BP dominance) are shown.

### Planktonic eukaryotic richness and ecological diversity in oligotrophic waters

**Figure 4:**
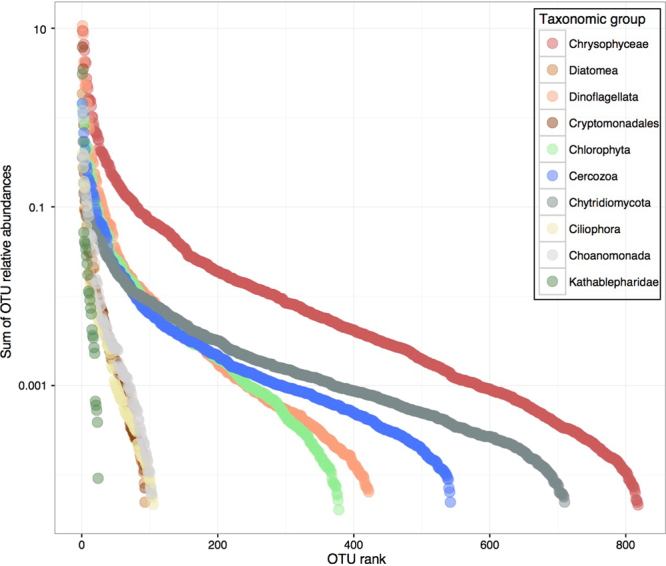
Rank abundance plot (log scale) of OTUs by taxonomic groups of interest. Cumulative sum of relative abundances determines OTU rank per group.

Chrysophytes, Chytridiomycota, Dinoflagellata, Cercozoa, and Chlorophyta showed the highest richness, Shannon diversity >1 per lake, and Berger-Parker dominance around 15% (Table 1). Lower richness and Shannon diversity (around 0.5) was observed in Kathablepharidae, Cryptomonadales, Diatomea, Ciliophora, and Choanomonada with Berger-Parker dominance >30%. The metacommunity patterns of gamma-diversity (rank-abundance curves) slightly diverge from the previous alpha-diversity picture. The gentler the slope of the curves, the greater the gamma-diversity (Fig. 4). Chytridiomicota, Chrysophyta, and Cercozoa were again the most diverse groups but the two heterotrophic groups showed more spatial beta-diversity than Chrysophyta. Chlorophytes and Dinoflagellata showed intermediate values and the diversity for the remaining groups was low, particularly, in Kathablepharidae.

**Figure 5:**
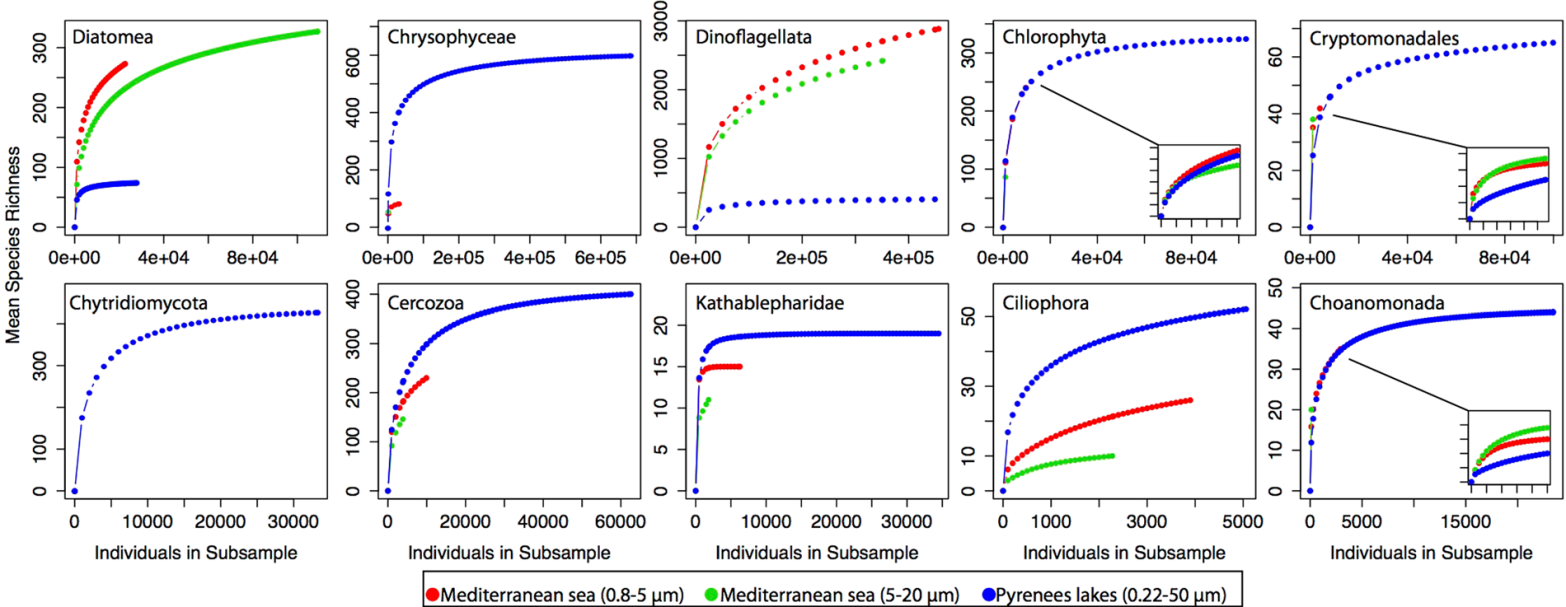
Rarefaction curves per taxonomic group of the Pyrenean pool (227 samples rarefied as a single pool) versus the Mediterranean Sea pool from the Tara Oceans project (11 samples per size fraction). Despite the variable number of samples, we equalized both pools by rarefying at equivalent sequencing depths per pool (see Supplementary information).

Interestingly, the freshwater Pyrenean metacommunity contained more diverse specific populations than the closest marine oligotrophic equivalent, surface waters of the Mediterranean Sea (Tara Oceans Project^16^) after normalizing to equivalent sequencing efforts (Fig. 5). This result was more apparent for Chrysophyceae, Cercozoa, Katablepharidae, and Ciliophora. Conversely, Diatomea and Dinoflagellata, appear to hold richer plankton populations in the Mediterranean Sea. Similar values were found for Choanomonada, and very few chytrids were reported in the Mediterranean dataset. In addition, higher beta-diversity was found for the regional freshwater study than in the marine surface samples across the Mediterranean (see more details in Supplementary information S2).

In general, the similarity between lake communities was significantly related to the geographic distance (Mantel test, Spearman method, r = 0.21, p = 0.001). In particular, this relationship was significant for Chrysophyceae (r = 0.16), Dinoflagellata, Ciliophora, and Cercozoa although the variance explained was low (Table 2). Therefore, we further analysed the diversity partition across the region considering catchments and sub-catchments. The mean beta-dispersion of basin and sub-basin communities was 0.53 (sd = 0.04) and 0.40 (sd = 0.07), respectively. The ANOVA of distances to group centroids was not significant for catchments (F = 1.89, p = 0.12). Conversely, the same analysis for sub-catchments was significant (F = 2.28, p < 0.001), and the *post hoc* Tukey HSD test showed that the taxonomic groups could be categorised into different levels of sub-basin beta-diversity (Table 2). The analysis highlighted the main heterotrophs with high beta-dispersion (i.e., Chytridiomycota and Cercozoa, ‘a’) from the main primary producers with low beta-dispersion (Chrysophyceae and Dinoflagellata ‘c’), and Kathablepharidae (‘d’) as the group with the lowest beta-dispersion. There was a positive correlation among the beta-dispersion of most of the groups per sub-basins (Fig. S1), being especially remarkable the high correlation and degree of significance (P < 0.001) of the Chytridiomycota with many of the other groups.

**Table 2:** Geographic relationships statistics among group communities.

Group	Mantel-r	Basin beta-dispersion (mean ± SD)	Sub-basin beta-dispersion (mean ± SD)	Tukey HSD test groups
Chytridiomycota	−0.02	0.66 ± 0.03	0.52 ± 0.07	a
Cercozoa	0.07*	0.63 ± 0.04	0.49 ± 0.08	ab
Chlorophyta	0.05	0.61 ± 0.07	0.49 ± 0.08	ab
Ciliophora	0.07**	0.64 ± 0.05	0.48 ± 0.11	ab
Diatomea	−0.02	0.64 ± 0.03	0.47 ± 0.11	abc
Choanomonada	0.01	0.60 ± 0.06	0.46 ± 0.12	bc
Cryptomonadales	−0.07	0.57 ± 0.07	0.45 ± 0.11	bc
Chrysophyceae	0.16**	0.56 ± 0.05	0.41 ± 0.10	c
Dinoflagellata	0.09**	0.56 ± 0.03	0.42 ± 0.09	c
Kathablepharidae	0.04	0.47 ± 0.05	0.32 ± 0.14	d

Mantel test results between group communities and geographic distances (*p < 0.01, **p < 0.001). Beta dispersion comparison per basin and sub-basin. Beta-dispersion was calculated per sub-basin when there were at least two lakes to compare. Statistical significance was assessed through *post-hoc* Tukey HSD for sub-basins: groups a–d are groups of analogous beta-dispersion patterns.

## Discussion

Historically, knowledge on protist assemblages from high mountain lakes was mainly based on ecological studies (e.g. Capblancq, 1972; Tilzer, 1972)^33,34^. They suspected a high diversity in some groups such as Chrysophyceae and Dinoflagellata, similarly as found in other oligotrophic systems (Nordic and Central European sites) by early taxonomical studies. Our study broadens this suspected high diversity and indicates a high degree of novelty, even in the case of Dinoflagellata with apparently closer culture counterparts especially for the most abundant OTUs. The comparison with the marine samples primarily highlights the Chrysophyceae richness in mountain lakes, whereas dinoflagellates are richer in the marine environment. Since chrysophytes lack carbon concentration mechanisms^35^, these are particularly suitable for these soft water environments, and we can interpret richness differences as an evolutionary divergence of the ecology between the two groups. This hidden high diversity in Pyrenean chrysophytes was already suspected based on 210 stomatocyst morphotypes found along 105 lakes^36^. The molecular survey carried out in the present study, suggests that the chrysophycean richness is at least four-fold larger. Interestingly, in classical treatises of Chrysophyceae morphospecies descriptions, the number raised to about one thousand (e.g., Starmach, 1985)^37^, though only a few of them have been cultivated. Chrysophyceae also includes heterotrophic organisms. Many of them are usually handled as *Spumella*-like organisms, and c. 50 Pyrenean OTUs could be assigned to this genus. A recent study indicates that *Spumella*-like flagellates are polyphyletic and that their lack of morphological differences can be seen as a convergence to a successful live form under certain ecological circumstances^38^. Mountain lakes, with a dark long ice-covered period^39^, may be a suitable environment for the genetic diversification of this life-form.

Dinoflagellates are abundant and diverse in mountain lakes, although less than in marine samples^16^. The high richness is particularly concentrated in small and unarmored forms (e.g. *Gymnodinium*). As is the case for Chrysophyceae, the molecular techniques provide a tool to investigate solidly the ecology of mountain dinoflagellates, which with the few exceptions of some *Peridimium* have remained elusive to traditional taxonomist. The result obtained for diatoms conformed to the established knowledge. Most of the diversity largely corresponds to benthic forms and, therefore, the modest contribution to total planktonic diversity does not come as a surprise. Moreover, as expected, the bulk of the OTUs corresponded to centric diatoms. The match with the cultured *Fragilaria nanana* agrees with the observation of this species in mixing epilimnia^40^. The group Cryptophyceae (both Cryptomonadales and Kathablepharidae) showed low diversity as compared with its high abundance and widespread occurrence. This feature is not something particular to mountain lakes but general for the group. The low morphological differentiation does not hide a high molecular diversity, although molecular taxonomy revealed additional variation^41^. Finally, we confirmed the presence of Haptophyta (Pavlovophyceae and Prymnesiophyceae) most of them closely related to cultured counterparts (e.g. *Chrysochromulina parva*), a group with little penetration in freshwaters and that had been only sceptically reported in mountain lakes^42^.

Molecular approaches provide taxonomic accuracy to the ecological studies, which up-to-now was only available to a few taxonomic specialists, as in the large variety of biflagellate Archaeplastida previously reported^3,42^ or ciliate species. The case of Ciliophora stands out since their morphological diversity is well known in at least one lake^2,42^. Combining metabarcoding and morphology of Ciliophora would allow straightforward understanding of their ecology and behaviour. Cultured references in general offered poor taxonomic quality, as in the case of non-flagellated Archaeplastida (e.g. Chlorococcales). But in some cases OTUs had close counterparts, as in Haptophyta or flagellated Archaeplastida. Molecular approaches can help discern between organisms with homologous morphologies. However the V9 region of the 18 rRNA gene lacks resolution to discern between species^10^. Furthermore, the degree of differentiation of this highly conserved region between organisms can vary between lineages, and its delimitation lies beyond the scope of this article. The fact that the same cultured strain was the CCM to several OTUs (e.g. *Gymnodinium*) or to marine genera, further confirms that the resolution of V9 18S rRNA region is low, and especially critical in the Alveolata group (see more details in Supplementary information). An effort to connect metabarcoding with morphological observations appears as a straightforward and fruitful way to follow. The wide range of monographs on freshwater Archaeplastida or Diatoms^21,43–45^; based on microscopical morphological observations could benefit of single-cell genomics^46^ or multi marker gene analysis to help fully disentangle a complex overlooked eukaryotic biosphere and their ecological dynamics. For example, if the correspondence between cyst types and Chrysophyceae barcodes is confirmed, the molecular techniques can provide more comparable ecological studies across sites. The cysts sedimentary record would enlighten a view of the population dynamics over millennia in paleoecological studies, so far mostly addressed with the diatom records.

### Unveiling unexpected heterotrophic diversity in oligotrophic freshwaters

The variety of heterotrophic flagellates in planktonic ecosystems has been traditionally underestimated^47^, particularly in mountain lakes^48^. Our results unveil an unexpected high heterotrophic diversity in several main groups. Further than the aforementioned *Spumella*-like organisms from Chrysophyceae, less richer phagotrophic flagellated groups, such as Choanomonada, Kathablepharidae or Bicosoecida^49^ also presented a higher diversity than expected. Chytridiomycota was the second richest group. The close relationship with cultured counterparts indicated that some of them may be pollen saprophytes. However, a vast richness remains with unknown roles. Chytridiomycota show the highest beta-dispersion, whose patterns per basin were significantly different to the main primary producers (Chrysophyceae and Dinoflagellata). This agrees with specialised saprophytic and parasitic roles^9^. The marked difference between the total species richness (gamma diversity) and the average richness per lake (alpha diversity) indicates a high specificity and punctual incidence in the communities as previously suggested^50^. However, only time series studies in a selected number of lakes can further provide the right answer to this hypothesis. The richness of Chytridiomycota in different aquatic systems is cropping out as more habitats are investigated with molecular techniques^51^. The low average richness per lake compared to the high metacommunity diversity suggests highly specific host interactions. The outstanding heterotrophic diversity is present also in Cercozoa (Rhizaria), whose role has been largely ignored, yet specimens were often observed in microscopic assessments. Parts of this diversity are benthic organisms such as the ameboid forms with organic or siliceous theca. However, flagellated forms (Cercamonadidae and Glissomonadida) spherical ameboid forms with filopodia, such as the Vampyrellidae, which are likely truly planktonic, also contributed with a substantial richness. Overall, and according to very recent findings in aquatic environments (see below), this trait is not exclusive of mountain lakes but probably an unseen key piece gearing planktonic food webs.

### Reconsidering planktonic food webs

Recent studies have been highlighting the relevance of the heterotrophic component in the marine protists diversity^16^ and biological interactions for the community dynamics^52^. The large diversity observed in the present investigation provides the building blocks for reconstructing mountain lake food webs. In these small lakes, the fuelling of the planktonic food web does not depend exclusively on its own primary production but also on organic materials supplied at the interfaces with the atmosphere, the benthic lake habitats, peri-lacustrine environments and terrestrial soils through direct and diffuse runoff^2,43,53^. The external loading of organic material enhances the heterotrophic character of the metacommunity by facilitating growth of saprophytic organisms releasing nutrients into the food webs (e.g. Chytridiomicota growing on pollen grains) and phagotrophic heterotrophs grazing on associated microbial communities. Indeed, the gamma diversity comparison with marine samples, from sites of similar primary productivity remarks the exacerbated saprophytic character of the lake communities. We may further wonder about the role of clearly terrestrial organisms, such as Dikarya or Zygomycota. Their high abundance and richness probably correspond to dispersal stages that constitute a source of food for protists and aquatic invertebrates^54^. In addition, the high richness of truly aquatic fungi or mycoplankton (e.g. Chytridiomycota) indicates a key role of parasitism on the lake plankton dynamics. Detailed studies relating host and Chytridiomycetes infections have focused on some key species (e.g. *Asterionella*^55^ or *Daphnia*^56^). Others have studied their implication on the proposed ‘mycoloop’^57^, which would sustain zooplankton populations under algal bloom conditions by increasing palatability^58^. How this loop would function under highly oligotrophic circumstances has high research potential. The increasing awareness of the role that Chytridiomycetes play in controlling host populations^59–61^ highlights parasitism in lake ecosystem dynamics as a key missing pathway as some time ago occurred with viral infections^62^. The latest molecular approaches provide solid foundations for reviewing the food web paradigm in oligotrophic freshwater lakes^9^ where parasitic groups are suspected to have high relevance^63^.

A large variety of parasites from all the main evolutionary branches were also abundant like in Stramenopiles (Labyrinthulomycetes, Peronosporomycetes, Hyphochytriales, Opalinata and *Blastocystis*), Alveolata (Apicomplexa, Perkinsidae and Syndiniales), and Opisthokonta (Blastocladiomycota, Ictyosporea, Myxozoa, Zoopagomycotina). Many of them may be present only as dispersal stages (e.g. zoospores and sporozoites). In any case, the large number of OTUs found demand more consideration of all these organisms in the lake ecosystem. Up to recently, some of the groups were mostly known from marine systems (e.g. Labyrinthulomycetes, Perkinsidae, Syndiniales). Perkinsidae have been found in freshwater only a few years ago^64^, and molecular surveys are increasingly reporting them^65^. These organisms can be parasites of algae, fish, bivalves and amphibians. Parasitism on benthic invertebrates and macrophytes may have a potential impact on the planktonic community by top-down interactions since littoral can be considered extending to the whole lake in many small mountain lakes. Parasites infecting protist, rotifers and planktonic crustaceans may have a direct influence on the planktonic food web (e.g. Chytridiomycota, Peronosporomycetes, Perkinsidae, Syndiniales, Zoopagomycotina). The stocking of Fish in mountain lakes throughout the world by humans may be highly relevant at this respect^66^. Thus parasites related to them (e.g. Peronosporomycetes, Perkinsidae, Syndiniales, Ichtyosporea, Myxozoa) may merit more insight in future studies, particularly those more virulent. As Chytridiomycota, some groups include a range of life forms; for instance, Blastocladiomycota, are zoosporic fungi that some are saprophytic on refractory materials (e.g., pollen, keratin, cellulose, chitin), grow in the submerged parts of marsh plants and may be specific parasites of nematodes, midges and crustaceans. Like LKM11, the ecological role of potentially basal fungal groups remains to be assessed in these oligotrophic lakes. In-depth studies are required but, whatever the specific roles that will come out, the conclusion that saprotrophic and parasitic interactions are central to the dynamics of the lake planktonic communities will probably remain.

### Data accessibility

Raw files for the genetic datasets are available in the NCBI SRA database under project id PRJNA413654.

## Electronic supplementary material

Supplementary Information
