# Supplementary material for: High planktonic diversity in mountain lakes contains similar contributions of autotrophic, heterotrophic and parasitic eukaryotic life forms
Source: Sci Rep. 2018 Mar 13;8:4457. doi: 10.1038/s41598-018-22835-3 (PMC5849755; doi:10.1038/s41598-018-22835-3)

SUPPLEMENTARY INFORMATION

High planktonic diversity in mountain lakes contains similar contributions of autotrophic, heterotrophic and parasitic eukaryotic life forms

Rüdiger Ortiz-Álvarez, Xavier Triadó-Margarit, Lluís Camarero, Emilio O Casamayor, and Jordi Catalan

Supplementary information S1. *Cultured references that matched with sequences of previously detected organisms (through morphology and microscopy observations)*

The highly conserved nature of the V9 18S rRNA region may preclude species classification. However, some CCMs confirm some previous observations in high mountain lake planktonic communities or indicate knowledge gaps of particular interest. At this respect, the number of Chlorophyta OTUs with close cultured counterparts was remarkable (115). About one-third of them were present in more than ten lakes. The results confirm that many elongate flagellated forms are already known (e.g., some *Chlamydomonas* and *Monomastix* species). In contrast, the CCMs for chlorophyte coccal forms are poorly taxonomically determined (e.g. *Chlorococcum* spp., *Chlorella* spp., and *Coccomixa* spp.). Abundant OTUs classified as Cryptophyceae matched cultures of *Cryptomonas marssonii* and, *Plagioselmis nannoplanctica*, among others. Some OTUs matched heterotrophic forms of the group such as *Goniomonas truncata* and several strains of *Chilomonas* that were present in about 20% and 10% of the lakes, respectively. The few OTUs from Haptophyta matched cultures of Pavlovophyceae species, and a widespread one in the lake dataset matched *Chrysochromulina parva* (Prymnesiophyceae).
Among the aquatic fungi, less than 10% of the Chytridiomycota OTUs matched cultured strains. Most of them related to *Rhizophydium*, *Rhizophlyctis* or *Rhizoclosmatium*. Ten of the OTUs were widespread (present in 20-30% of the samples) but not particularly abundant. Some of the related cultures correspond to species growing on pine pollen (e.g. *Rhizophlyctis harderi* and *Kappamyces laurelensis*).
Cercozoa – the group with the highest diversity among phagotrophic heterotrophs in our samples – includes several life-forms with pseudopodia that correspond to the main phylogenetic branches (Cavalier-Smith, 2003). The most common OTU (20% samples) matched cultures of the thecate *Rhogostoma schuessleri* (Cl. Thecofilosea). Some flagellated forms were also common; OTUs matching *Cercomonas rotunda* and *Paracercomonas crassicauda* cultures were present in about 5% of the samples among other species of the same genera. The species with silica plates (Imbricatea) were less frequent (< 1%) but showed matches with a large diversity of genera (*Allas*, *Assulina*, *Peregrinia*, *Euglypha*, *Spongomonas*).

The SAR groups largely differed regarding culture references. Ciliophora showed a low number of CCM and only an OTU matching a cultures of *Rimostrombidium lacustris* (20% of samples) was relevant. On the other hand, Dinoflagellata showed CCM for many of

the most ubiquitous and abundant OTUs. Several OTUs matching the same culture of an undetermined *Gymnodynium* were ubiquitous and indicate a potentially large hidden variability in these abundant small dinoflagellates. Similarly, may occur with *Woloszynskia*-like species. The most abundant OTU in the complete survey belongs to this group. Only two OTUs closely matched cultured strains *W. leopoliensis* and *W. pascheri*, present in 10 and 2% of the samples, respectively. Among armoured forms, *Peridinium inconspicuum* and *P. cinctum* cultures are close to OTUs present in 3 and 10% of the lakes. Within Chrysophyceae only 51 OTUs had a CCM reference, despite being the richer protist group in the lake samples. OTUs related to cultures of several *Dinobryon* species, conspicuous forms with large colonies of cells with theca, were regularly present in many lakes. Among the OTUs with high occurrence, several matched cultures of flagellated forms with siliceous scales (Synurales), which are currently split from the Chrysophyceae into a separate class (Synurophyceae). OTUs matching *Mallomonas* species were present in more than 50% of the lakes. Most Chrysophyceae are planktonic, therefore, the appearance in a few lakes of OTUs matching benthic forms, rarely reported in field surveys, are worth to mention (e.g., *Chrysochaete, Chrysonephele* and *Chrysocapsa*). In contrast, the naked flagellated autotrophic forms, which are extremely rich in these lakes only showed a few cultured matches. OTUs matching cultured heterotrophic Chrysophycean forms were abundant. Unfortunately, the taxonomy of the cultures was poor; most of them are simply labelled as "*Spumella*-like". Some diatom OTUs showed a high matching with cultured referents; unfortunately the latter were poorly determined. They mostly belonged to Mediophyceae, centric diatoms that are truly planktonic. An exception to the general taxonomic uncertainty was *Fragilaria nanana*, with a 100% match with one of the OTUs and present in 25% of lakes.

Supplementary information S2: *Comparison of Pyrenean freshwater and Mediterranean sea surface samples*

Context

The comparison between freshwater and marine planktonic systems of similar primary production can enlighten the general ecological constraints and evolutionary trajectories that have been shaping planktonic communities. For example, Pyrenean lakes and Mediterranean open waters show roughly a similar primary production, about 100±50 g C m^{-2} yr^{-1} (Catalan *et al.*, 2006; Siokou-Frangou *et al.*, 2010). Species of oligotrophic freshwater and marine planktonic assemblages have been evolving in ecosystems of similar flow of autochthonous carbon but contrasting environmental conditions, the size of the system and inputs of subsidiary organic matter from neighbouring ecosystems. Therefore, one may expect both common and contrasting features between oligotrophic marine and freshwater planktonic communities. We compared the diversity patterns found in our survey with those of the Tara Oceans expedition in the Mediterranean system (Vargas *et al.*, 2015). Commonalities and major differences provided elements for discussing on how the common oligotrophy but the different environmental setting may have been influencing the evolutionary history and the ecological configuration of these planktonic systems.

Specific methods

We downloaded a set of 22 marine libraries of the V9 18S rRNA region, from the Tara Oceans expedition (Vargas *et al.*, 2015) to compare the richness of different groups. We chose subsurface (mixed layer) Mediterranean samples, because of the similar primary productivity status to the lakes studied (Siokou-Frangou *et al.*, 2010). We selected samples from 0.8-5 µm and 5-20 µm sizes, which despite dismissing organisms >20 µm was a close equivalent to the plankton size typically found in the Pyrenean lakes (0.22-50 µm) (Sarmento *et al.*, 2015).

Data filtering and classification was carried out with the same parameters as for the Pyrenean samples, except filtering to a read length of 163. For simplicity, the comparison was made using a single classification method (RDP classifier). To minimise information loss and potential sampling strategy bias, we established a compromise between the number of samples and the sequencing depth for each sequence pool. A total of 227 Pyrenees samples were rarefacted to 10,000 sequences (2,270,000 sequences). And, in accordance, 11 Tara samples (for each filter size) were rarefacted to 200,000 sequences (2,200,000 sequences) in order to equalize sequencing effort between studies. Good's coverage was 0.77 and 0.74 for the marine pools and 0.94 for the Pyrenean one, indicating a better capture of the regional diversity in the latter pool, but still adequate for our goal. We calculated richness and Shannon diversity in the marine samples for alpha diversity comparison with the Pyrenean samples.

Comparison results

The diversity comparison between the Pyrenean lakes and the Mediterranean Sea samples from the Tara Oceans expedition cannot be straightforward because of the size

partition in the marine survey and its much larger number of samples and sequences. Therefore, we concentrated in the main taxonomic groups of the Pyrenean lakes and compared them with the rarefied results of the two ocean size-filters (Suppl. Table 1, Figure 5). Comparing individual samples, the average richness and Shannon diversity (H') per group were higher in the marine samples than in the freshwater ones (Suppl. Table 1) with a few exceptions. Chrysophyceae showed more species per lake (average, 68) than per marine spot (27, adding the two filters), although H' was slightly higher in the marine samples of the small filter (0.8-5 μm). In general, this filter showed much higher H' values than the 5-20 μm filter, except for diatoms. The richness of Ciliophora and Kathablepharidae was similar between the freshwater and marine samples, although H' was more than twice higher in the marine samples; indicating a more evenly distributed abundance of the species for these groups in the sea. Chytridiomicota richness and H' were similar to Chlorophyta and Cercozoa in the freshwater samples (about ten species per sample on average) whereas they were absent in the rarefied samples of the Tara Oceans expedition. Beyond these exceptions, the other groups were richer and more diverse in the marine samples than in the mountain lake samples. The difference was particularly outstanding for Dinoflagellata, with an order of magnitude more species in both marine filters (ca. 300 species per sample) than in the freshwater ones (ca. 30) although this group places second in the lake's richness rank.

Table S1: Average richness and Shannon diversity for each group for Mediterranean Sea samples (size fractions '0.8-5 μM' and '5-20 μM') and Pyrenees samples (a single size fraction Med (0.22-20 μM), after rarefaction to 10000 all samples and transform them to relative abundances. OTU classification into taxonomic groups was done using RDP classifier only.

	Richness			H'		
Group	Pyrenees	Med (0.8-5 μM)	Med (5-20 μM)	Pyrenees	Med (0.8-5 μM)	Med (5-20 μM)
Chytridiomycota	10	0	0	1.42	0.00	0.00
Ciliophora	2	2	2	0.33	0.65	0.62
Kathablepharidae	6	7	2	0.45	1.66	0.59
Choanomonada	2	6	1	0.28	1.66	0.34
Cryptomonadales	6	10	2	0.55	1.89	0.50
Diatomea	4	23	31	0.55	2.37	2.31
Chlorophyta	10	17	6	1.15	2.46	1.05
Cercozoa	11	19	6	1.37	2.57	1.19
Chrysophyceae	68	25	2	2.17	2.80	0.62
Dinoflagellata	30	321	216	1.44	4.50	4.12

The high richness and diversity of Dinoflagellata and Diatomea per marine sample remain when the overall metacommunity diversity is compared amalgamating all the samples and rarefying per individuals (Figure S2). However, the situation changes for the other groups. The freshwater metacommunity results more diverse for

Chrysophyceae, Chlorophyta, Cryptomonadales, Cercozoa, Katablepharidae and Ciliophora, and similar for Choanomonada (Figure S2). This difference between average features per sample (alpha diversity) and the patterns of the overall ensemble (gamma diversity) indicates more different assemblages between the mountain lake samples than between the marine ones. This indicates higher beta-diversity in the freshwater studied area than in the marine samples.

References

Catalan J, Camarero L, Felip M, Pla S, Ventura M, Buchaca T, *et al.* (2006). High mountain lakes: extreme habitats and witnesses of environmental changes. *Limnetica* **64**: 123–145.

Cavalier-Smith T. (2003). Protist phylogeny and the high-level classification of Protozoa. *Eur J Protistol* **39**: 338–348.

Sarmento H, Casamayor EO, Auguet JC, Vila-Costa M, Felip M, Camarero L, *et al.* (2015). Microbial food web components, bulk metabolism, and single-cell physiology of piconeuston in surface microlayers of high-altitude lakes. *Front Microbiol* **6**: 1–12.

Siokou-Frangou I, Christaki U, Mazzocchi MG, Montresor M, d'Alcala MR, Vaque D, *et al.* (2010). Plankton in the open Mediterranean Sea: a review. *Biogeosciences* **7**: 1543–1586.

Vargas C De, Audic S, Henry N, Decelle J, Mahé F, Logares R, *et al.* (2015). Eukaryotic plankton diversity in the sunlit ocean. **348**: 1–12.

Supplementary Figure S1. Correlations between taxonomic groups of interest based on average beta-dispersion per sub basin. Darker color indicates higher correlations. Only correlation values with strong significance ($p < 0.001$) are displayed.

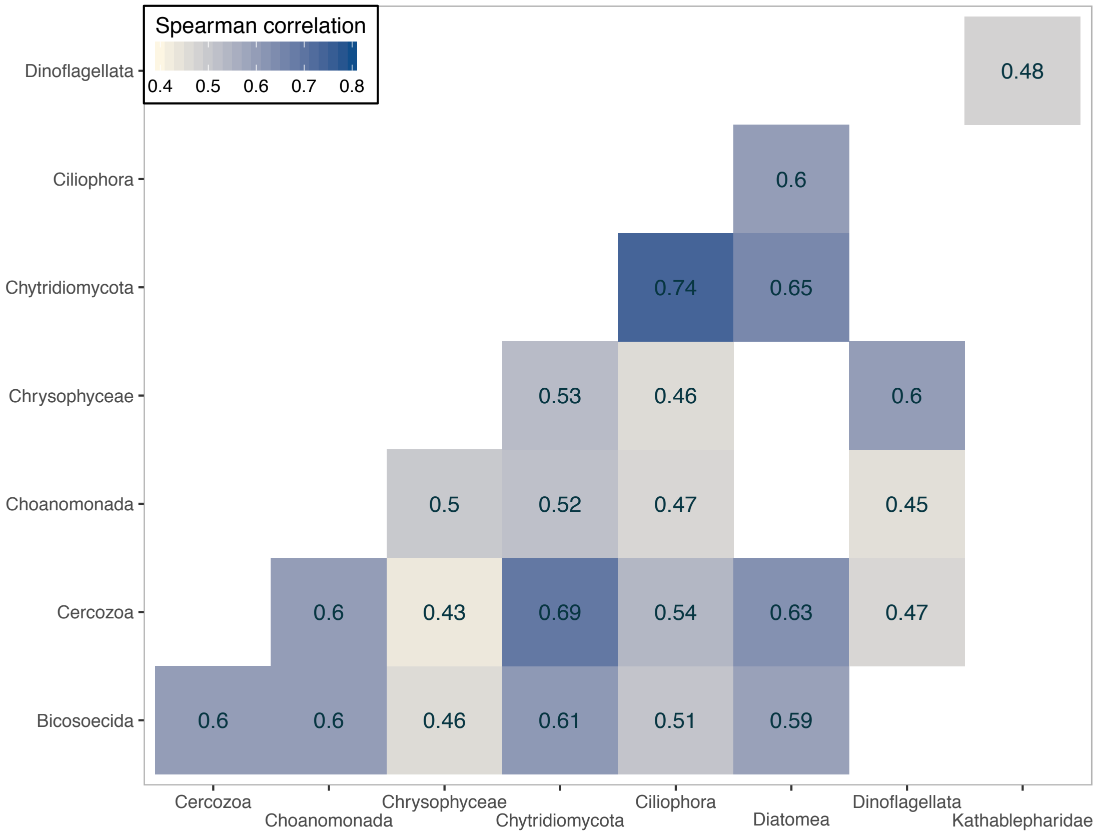

Supplement: Supplementary file 1 — Supplementary Information [file 41598_2018_22835_MOESM1_ESM.pdf]
